# Adenomyomectomy with preconceptional transabdominal cervicoisthmic cerclage for transvaginal cerclage failure

**DOI:** 10.3389/fsurg.2025.1484120

**Published:** 2025-06-13

**Authors:** Kyong-No Lee, So-Yoon Park, Ga-Hyun Son, Keun-Young Lee

**Affiliations:** ^1^ Department of Obstetrics and Gynecology, Chungnam National University School of Medicine, Chungnam National University Hospital, Daejeon, Republic of Korea; ^2^ Division of Maternal-Fetal Medicine, Department of Obstetrics and Gynecology, Hallym University College of Medicine, Kangnam Sacred Heart Hospital, Seoul, Republic of Korea; ^3^ Institute of New Frontier Research Team, College of Medicine, Hallym University, Chuncheon, Republic of Korea

**Keywords:** adenomyosis, laparotomy, myometrium, pregnancy outcome, preterm birth

## Abstract

**Background:**

Despite evidence of adverse pregnancy outcomes associated with adenomyosis, there are no established strategies for risk assessment, stratification, or prevention of these potential complications. This study aimed to describe and evaluate the technique of adenomyomectomy with preconceptional transabdominal cervicoisthmic cerclage (TCIC) in high-risk women with an unfavorable history of previous preterm birth and/or second trimester miscarriage after transvaginal cerclage.

**Methods:**

Eligible patients had adenomyosis, confirmed by ultrasound or biopsy, and a history of second trimester miscarriages or preterm deliveries before 28 weeks despite transvaginal cerclage. All patients underwent adenomyomectomy followed by preconceptional placement of TCIC. Their obstetric and gynecologic histories, surgical outcomes, and pregnancy courses were retrospectively reviewed. Of the 85 patients who underwent this procedure at our facility over a 10-year period, we report the outcomes of 17 patients with antenatal care and delivery records available at our hospital.

**Results:**

Seventeen patients were included in the final analysis. Only one patient delivered before 34 weeks, and six between 34 and 37 weeks. One patient had three successful pregnancies following the procedure.

**Conclusions:**

Adenomyomectomy with preconceptional TCIC may be a viable treatment option for patients with adenomyosis who wish to have a successful pregnancy after a previous preterm delivery or miscarriage.

## 1 Introduction

Adenomyosis is a medical condition characterized by ectopic endometrial glandular tissues and interstitium in the myometrium (1). It is a rare clinical diagnosis but a common pathological finding in hysterectomy specimens (2). Considering the recent trend of delayed childbearing and the requirements for assisted reproductive procedures, the rate of diagnosis of adenomyosis has increased (3). Studies have reported that gravid women with adenomyosis are at an increased risk of infertility, spontaneous preterm birth, premature rupture of membranes, and other pregnancy-related complications (4–7). In addition, according to a recent study by Rees et al. (8), women with histologically confirmed adenomyosis have a higher prevalence of adverse obstetric outcomes. Despite evidence of adverse pregnancy outcomes associated with adenomyosis, there are no established strategies for risk assessment, stratification, or prevention of these potential complications. Although total hysterectomy is the preferred treatment for adenomyosis, uterus-conserving surgery has recently gained attention, particularly among female patients who marry later in life and desire to preserve their fertility (9). Adenomyomectomy was first introduced as a conservative surgery by Hyams (10) in 1952, and early surgical methods used wedge resection and suturing, resulting in frequent recurrence and occasional serious complications, such as uterine rupture (11–13). Since then, various surgical methods have been introduced to reduce recurrence and complications, including both laparotomy and laparoscopic approaches (14, 15).

Classically, cervical insufficiency is a diagnosis based on an obstetric history of recurrent second- or early third-trimester foetal losses following painless cervical dilation, prolapse or rupture of the membranes, and expulsion of a live fetus despite minimal uterine activity (16). Cerclage is a treatment for women at risk of pregnancy loss due to cervical insufficiency. Transvaginal cerclage (TVC) is the most common first-line treatment, as it is technically easier to perform and can be removed close to term to allow for a normal delivery. However, approximately 13% of pregnancies in women with cervical incompetence (historical indications) treated with elective TVC will not be successful and will result in the delivery of pre-viable infants despite intervention (17). Transabdominal cervicoisthmic cerclage (TCIC) is indicated when TVC is extremely difficult to place in patients with anatomical problems (e.g., trachelectomy, recurrent loop electrosurgical excision procedures, and congenital extremely short cervix) or a history of unsuccessful TVC, defined as previous placement of history- or ultrasound-indicated cerclage and subsequent singleton delivery before 28 0/7 weeks of gestation (18). However, even after TVC or TCIC, adenomyosis is associated with preterm birth. According to Song et al. (19), maternal adenomyosis is linked to preterm birth before 34 weeks of gestation, even among women who undergo TCIC.

Herein, we describe a treatment solution for complex cases of adenomyosis wherein TVC may be ineffective. The features of our adenomyomectomy with preconceptional TCIC technique to prevent preterm delivery are presented, along with the outcomes of a series of patients who underwent treatment with this technique.

## 2 Materials, equipment and methods

**Figure 1:**
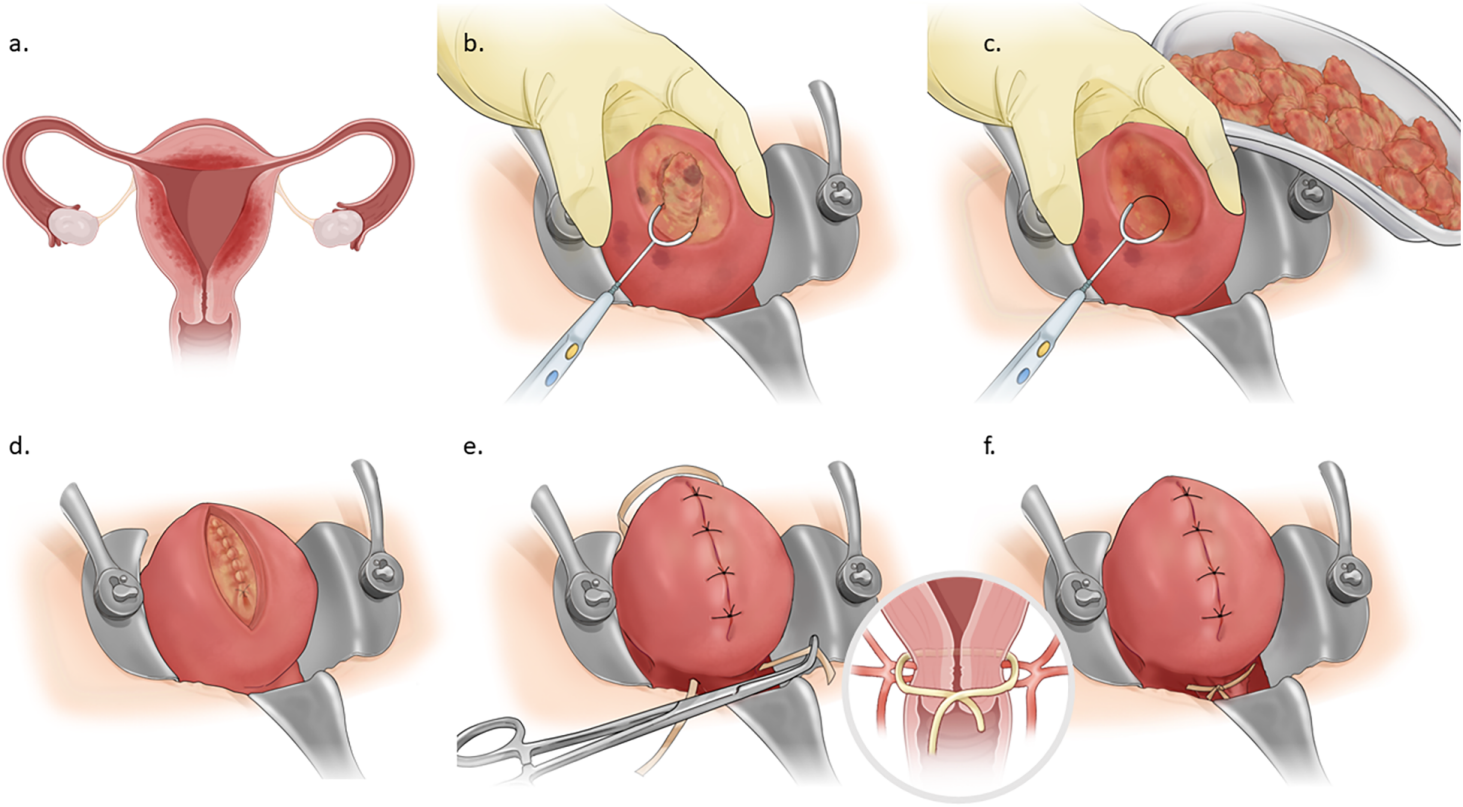
Schematic illustration of adenomyomectomy with preconceptional transabdominal cervicoisthmic cerclage. **(A)** Uterus with diffuse adenomyotic changes. **(B)** Longitudinal incision of the uterine wall. **(C)** Excision of adenomyotic tissue. **(D)** Uterine wall closure. **(E)** Mersilene tape insertion through avascular tunnel. **(F)** Uterus after adenomyomectomy with preconceptional transabdominal cervicoisthmic cerclage.

This study is a retrospective case series of patients who underwent adenomyomectomy with preconceptional TCIC for a history of failed TVC and confirmed adenomyosis. Data on 85 patients who had delivered between May 2011 and March 2021 and later underwent preconceptional adenomyomectomy with TCIC at the Hallym University Kangnam Sacred Heart Hospital in Seoul between June 2010 and April 2020 were obtained. The indications for adenomyomectomy and preconceptional TCIC were as follows: (1) TVC was performed during a previous pregnancy, but the patients delivered before 28 weeks or experienced second trimester miscarriages despite previous transvaginal cerclage, and (2) adenomyosis was confirmed using sonography and biopsy (Figure 1). This study was approved by the ethics committee of Hallym University Kangnam Sacred Heart Hospital (approval number: 2022-09-019; date of approval: September 28, 2022). The need for informed consent was waived because of the retrospective nature of the study.

**Figure 2:**
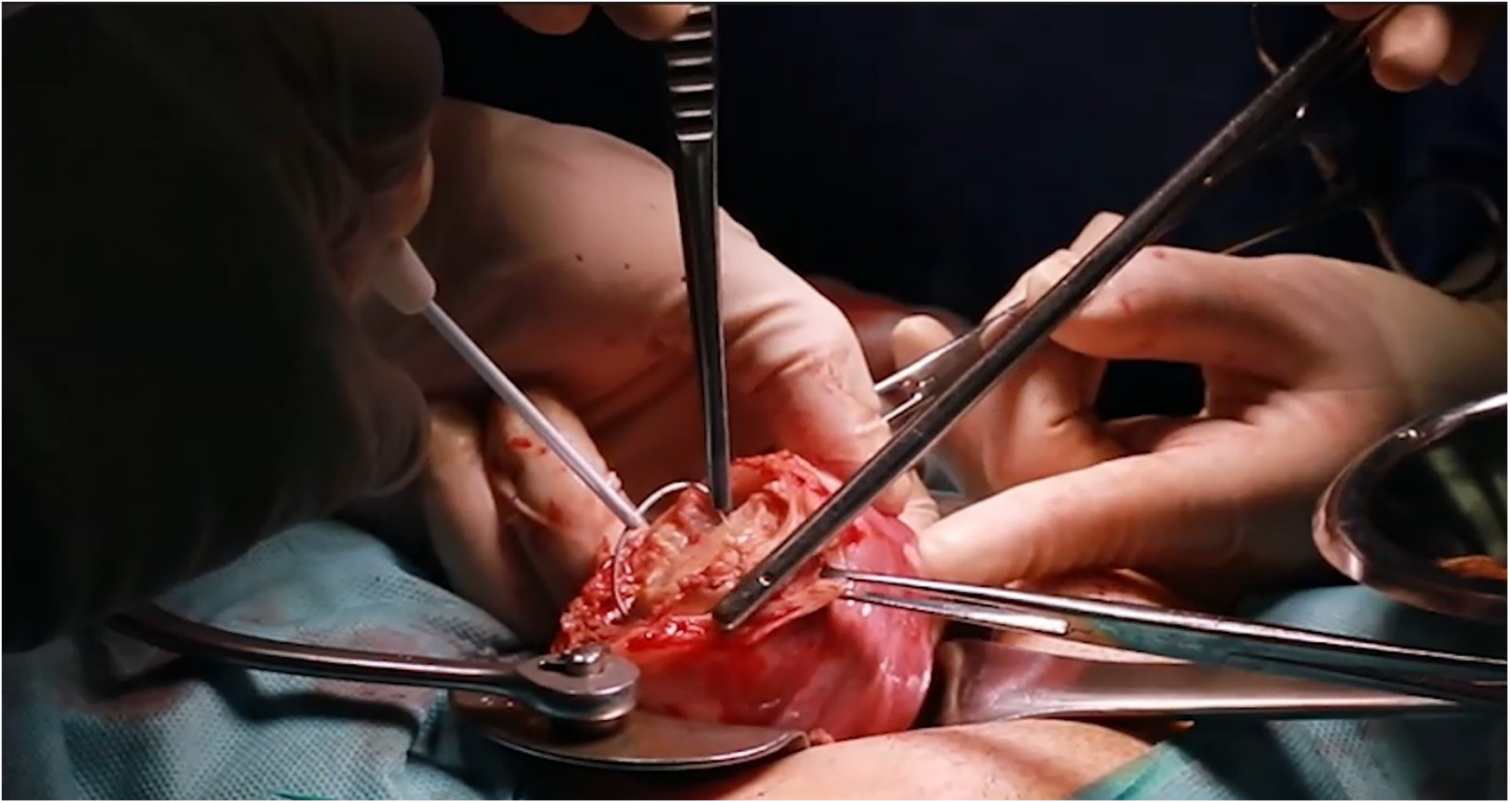
Representative intraoperative photographs of adenomyotic lesion palpation and excision.

**Figure 3:**
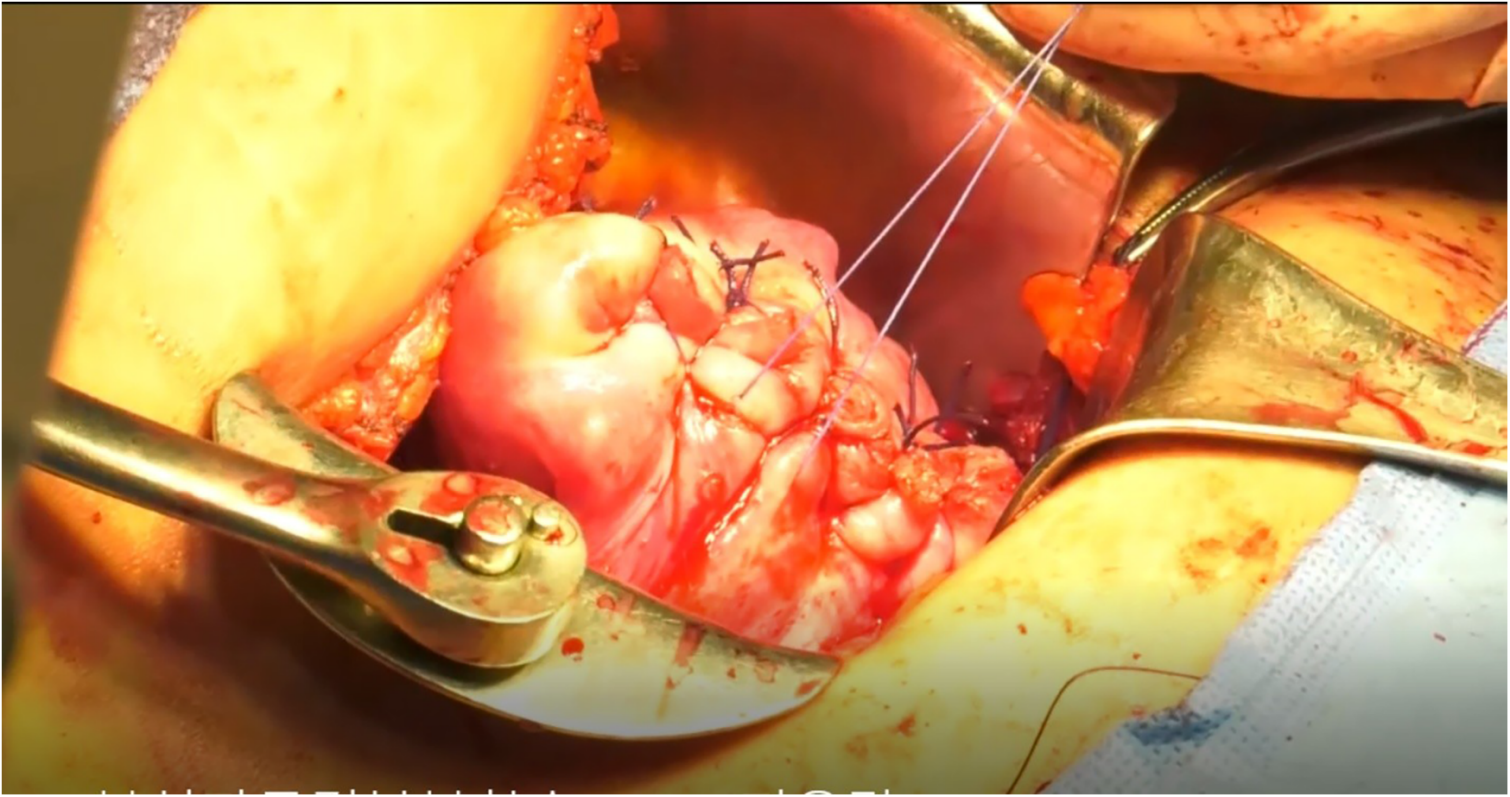
Representative intraoperative photographs of uterine wall closure.

**Figure 4:**
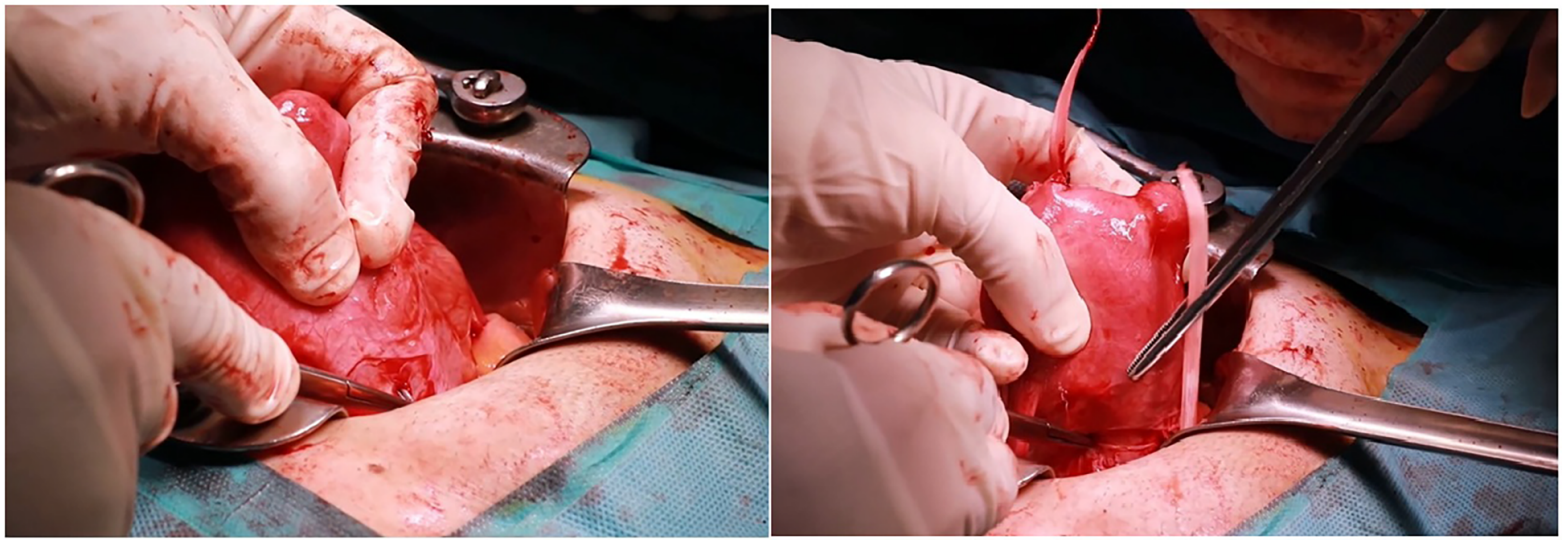
Representative intraoperative photographs of Mersilene tape insertion through the avascular tunnel.

**Figure 5:**
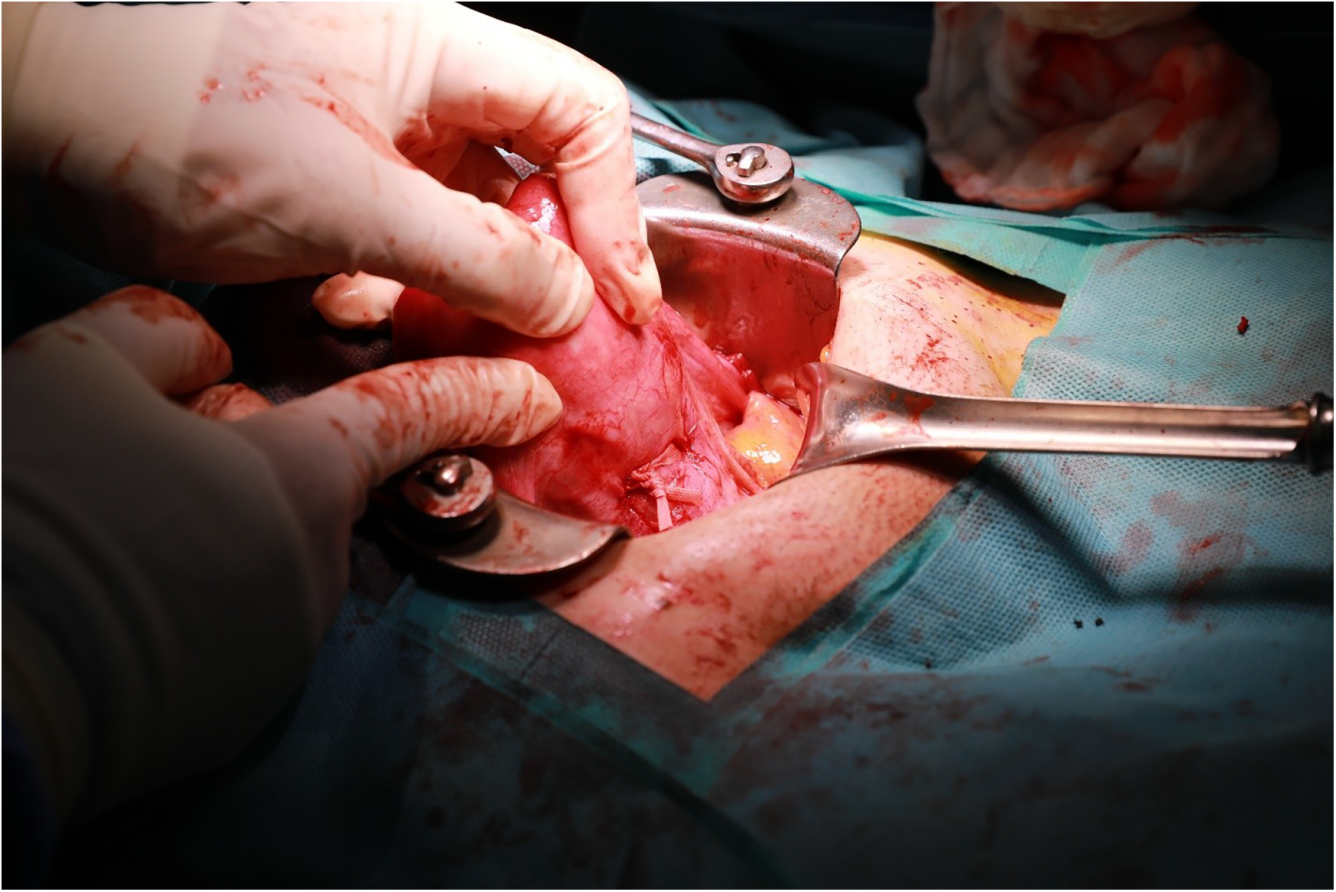
Representative intraoperative photographs of uterine repositioning and abdominal closure.

Laparotomy is performed, and 20ml of 100-fold diluted vasopressin is injected into the uterus, as illustrated in Figure 1. The area of adenomyosis detected on ultrasound is palpated, and a longitudinal incision is made (Figure 1A). The segment of the uterine wall affected by adenomyosis is removed using an electrosurgical loop. The area suspected to be adenomyosis is confirmed by palpation and resected using an electrosurgical loop to preserve as much normal myometrium as possible (Figures 1B,C, 2). The uterine wall is then closed using absorbable sutures (Figures 1D, 3). Following the adenomyomectomy, the cervicoisthmic region is exposed through sharp and blunt dissection of the vesicouterine peritoneum. The uterine vessels are laterally displaced to confirm the avascular space. The avascular region is perforated with a right-angle clamp, and a 5 mm Mersilene tape (Ethicon, Somerville, NJ, USA) is inserted through the tunnel in the anterior to posterior direction and tied anteriorly (Figures 1E, 4). After ensuring haemostasis, the uterus is placed back into the pelvic cavity, and the abdominal layers are closed (Figures 1, 5).

## 3 Results

Between May 2011 and March 2021, 85 patients underwent adenomyomectomy with preconceptional TCIC at our institution. All patients included in this study underwent surgery by the same surgical team at a single tertiary referral center, following a standardized protocol for both adenomyomectomy and TCIC placement. Despite variations in prior obstetric history, the surgical technique was consistent across all cases to minimize variability in outcomes. The mean age of patients who underwent surgery was 35.5 ± 3.6 years. Of these patients, 24, 21, and 14 had 1, 2, and at least 3 s trimester miscarriages or preterm deliveries, respectively, at <24 weeks. Twenty-two patients had one previous delivery each between 24 and 32 weeks, 10 had 2 previous deliveries each, and 2 had 3 or more previous deliveries each. Among these 85 women, 17 patients had antenatal care and delivery records available for review at our hospital. The outcomes of the 59 patients who did not deliver babies in our hospital or who did not have subsequent pregnancies were not assessed. The outcomes of nine patients with incomplete medical records were also not assessed. Table 1 shows the outcomes of the 17 patients. Only one patient delivered before 34 weeks, and six patients delivered between 34 and 37 weeks. One patient delivered three times.

**Table 1:** Outcomes of 17 patients who underwent adenomyomectomy with preconceptional transabdominal cervicoisthmic cerclage.

Case no.	No. of previous cerclage failures	Gestational age, weeks			Indication of delivery	Birth weight, g	Apgar score, 1 and 5 min	Remarks
		Previous cerclage	Previous delivery	Delivery after adenomyomectomy with preconceptional transabdominal cerclage				
1	1	19.2	19.6	39.0	Elective c/sec	2,700	10/10	GDM
2	1	22.2	22.5	39.0	Elective c/sec	3,100	8/10	
3	1	16.2	19.1	38.4	Elective c/sec	3,680	10/10	
4	2	20.3	28.4	35.5	PTL	2,980	6/8	Jaundice
		14.0	14.4					
5	1	15.0	20.0	38.2	Elective c/sec	3,630	9/10	GDM
			23.0					
6	2	23	28	38.2	Elective c/sec	3,670	8/9	
		13	17					
7	2	15.4		38	Elective c/sec	4,150	9/10	LGA, jaundice, polycythemia,
		−>22.3 (repeat)	23.4					
8	1	18.5	25.1	38.2	Elective c/sec	2,940	8/9	
9	1	15.1	24.6	36.5	PTL	3,240		Jaundice
10	1	15.2	17.3	37.6	Elective c/sec	2,650	10/10	
11	1	19.0	24.1	38.6	Elective c/sec	3,330	10/10	GDM
12	2	20.0	21.0	36.4	Elective c/sec	2,960	10/10	Transient tachypnea, PFO,
		13.1	18.5					Hyperbilirubinemia
13	2	25.0	27.0	38.4	Elective c/sec	2,780	9/10	Jaundice
		12.0	19.0	38.3	Elective c/sec	3,020	9/10	Both hydronephrosis,
				38.2	Elective c/sec	3,250	10/10	r/o hydrocele
14	1	13.2	20.1	36.4	Elective c/sec	2,120, 1,700	9/10, 9/10	Dichorionic diamniotic twin
15	1	12.0	18.6	36.3	Severe PE	2,610	8/10	
16	1	16.5	22.2	35.6	Severe PE	2,790	9/10	GDM
17	1	12.4	19.0	32.6	Severe PE	1,780, 1,100	7/8, 4/7	Dichorionic diamniotic twin, Discordant twin

c/sec, cesarean section; GDM, gestational diabetes mellitus; LGA, large for gestational age; PFO, patent foramen ovale; PTL, preterm labor; PE, Preeclampsia.

## 4 Discussion

Adenomyomectomy with preconceptional TCIC is a viable treatment option for patients who desire to conceive after a previous preterm delivery. This surgical technique is important, as it can benefit women who have experienced second trimester miscarriages or preterm deliveries before 28 weeks due to TVC failure caused by adenomyosis. Recent studies have shown that adenomyosis is linked to adverse perinatal outcomes, such as higher rates of spontaneous preterm delivery, preterm pre-labor rupture of membranes, preeclampsia, caesarean delivery, postpartum hemorrhage, placenta previa, and placenta accreta spectrum (4, 5, 20–25). According to Song et al. (19), maternal adenomyosis can, to some extent, be considered a predictor of delivery outcomes after TCIC. Based on the findings of previous research, adenomyomectomy has emerged as a potential approach to prevent preterm birth (26). It is not a standard treatment for diffuse adenomyosis because borderless adenomyosis tissue invades the uterine myometrium, resulting in inaccurate complete excision of the affected area, given that the removal of such tissue is always accompanied by excision of normal uterine muscle tissue (27, 28). Kwack et al. (3) reported that pregnant women who undergo adenomyomectomy can achieve safe perinatal outcomes under close monitoring for preterm labor and surveillance for catastrophic pregnancy-related complications. Furthermore, a few studies reported improved pregnancy outcomes after adenomyomectomy, even in cases of diffuse adenomyosis, suggesting that the surgery is a conservative and effective treatment method for adenomyosis (9, 26, 29, 30).

Several studies have explored the efficacy of TCIC, both during pregnancy and in the preconceptional period. While TCIC performed during pregnancy may be associated with higher surgical complexity and potential obstetric risks, preconceptional placement allows for optimal anatomic exposure and precise tape positioning. Moreover, various studies reported that women who underwent this procedure before conception were more likely to deliver after 34 weeks of gestation. In such studies, there were fewer preterm deliveries in the pre-pregnancy group and higher rates of surgical complications, including quantified blood loss of >500 ml, in the during-pregnancy group (31–33). Research comparing laparoscopy and laparotomy has shown similar results (34, 35). In a large systematic review, Burger et al. (34) found no difference in blood loss, operative time, or length of hospital stay when laparoscopic and laparotomy abdominal cerclage placements were compared. A more recent systematic review by Hulshoff et al. (35) revealed that laparoscopic abdominal cerclage placement was associated with less blood loss and a shorter hospital stay than laparotomy abdominal cerclage. However, laparotomy remains a practical approach, especially when performed in conjunction with adenomyomectomy, as in our study.

As mentioned earlier, all patients included in this study had undergone TVC but subsequently experienced second-trimester miscarriage or preterm delivery before 28 weeks of gestation, fulfilling the indications for TCIC. Although all cases met the inclusion criteria of prior TVC failure and confirmed adenomyosis, the etiology of cerclage failure may have been multifactorial. Due to the retrospective nature of the study, consistent data on cervical length trends, microbiological findings, and inflammatory markers were not available. Future studies should incorporate these variables to better elucidate the underlying causes of TVC failure and guide tailored treatment strategies. Based on the present findings, adenomyomectomy with preconceptional TCIC may be considered a surgical option in women with suspected adenomyosis who experience recurrent pregnancy loss or preterm birth before 28 weeks despite TVC.

The present study has several strengths. It focuses on a well-defined high-risk population with documented adenomyosis and prior failed transvaginal cerclage. Additionally, the surgical procedure was standardized, and the cases were managed at a single tertiary center, which reduces variability. However, the small number of patients (*n* = 17) is a significant limitation. The retrospective design and lack of a control group also limit the generalizability of our findings.

In conclusion, our findings suggest that adenomyomectomy with preconceptional TCIC may be a promising option for women with adenomyosis and a history of failed transvaginal cerclage. Future studies should focus on larger, multicenter prospective cohorts and investigate the impact of adenomyosis severity, lesion localization, and myometrial integrity on cerclage efficacy. Further investigation is also warranted to evaluate long-term reproductive outcomes and potential risks, such as uterine rupture in subsequent pregnancies.

## Data Availability

The original contributions presented in the study are included in the article/Supplementary Material, further inquiries can be directed to the corresponding author.
